# Do Muscle Synergies Improve Optimization Prediction of Muscle Activations During Gait?

**DOI:** 10.3389/fncom.2020.00054

**Published:** 2020-07-10

**Authors:** Florian Michaud, Mohammad S. Shourijeh, Benjamin J. Fregly, Javier Cuadrado

**Affiliations:** ^1^Laboratory of Mechanical Engineering, University of La Coruña, Escuela Politecnica Superior, Ferrol, Spain; ^2^Rice Computational Neuromechanics Laboratory, Rice University, Houston, TX, United States

**Keywords:** static optimization, synergies, gait, muscle forces, EMG validation

## Abstract

Determination of muscle forces during motion can help to understand motor control, assess pathological movement, diagnose neuromuscular disorders, or estimate joint loads. Difficulty of *in vivo* measurement made computational analysis become a common alternative in which, as several muscles serve each degree of freedom, the muscle redundancy problem must be solved. Unlike static optimization (SO), synergy optimization (SynO) couples muscle activations across all time frames, thereby altering estimated muscle co-contraction. This study explores whether the use of a muscle synergy structure within an SO framework improves prediction of muscle activations during walking. A motion/force/electromyography (EMG) gait analysis was performed on five healthy subjects. A musculoskeletal model of the right leg actuated by 43 Hill-type muscles was scaled to each subject and used to calculate joint moments, muscle–tendon kinematics, and moment arms. Muscle activations were then estimated using SynO with two to six synergies and traditional SO, and these estimates were compared with EMG measurements. Synergy optimization neither improved SO prediction of experimental activation patterns nor provided SO exact matching of joint moments. Finally, synergy analysis was performed on SO estimated activations, being found that the reconstructed activations produced poor matching of experimental activations and joint moments. As conclusion, it can be said that, although SynO did not improve prediction of muscle activations during gait, its reduced dimensional control space could be beneficial for applications such as functional electrical stimulation or motion control and prediction.

## Introduction

Knowledge of muscle forces during human movement could elucidate basic principles of human motor control (Pierrynowski and Morrison, 1985), facilitate assessment of pathological movement and diagnosis of neuromuscular disorders, and improve estimation of the loads experienced by diseased or injured joints (Hardt, 1978). Because *in vivo* measurement of muscle force is invasive and impossible for some muscles, computer modeling has become a commonly used alternative approach (Nagano et al., 2005). However, because more muscles than degrees of freedom (DOFs) exist in the human musculoskeletal system, an infinite number of recruitment patterns are possible mathematically. This problem is often referred to as the muscle redundancy problem (Damsgaard et al., 2006) or force-sharing problem (Dul et al., 1984).

The muscle redundancy problem is commonly solved by an inverse-dynamics optimization method called static optimization (SO) (Crowninshield, 1978; Ambrósio and Kecskeméthy, 2007; Shourijeh et al., 2017), which considers muscle activations as if each muscle was activated independently. However, recent studies have demonstrated that the central nervous system (CNS) appears to use muscle synergies to simplify neural control of movement by coupling muscle activations together (Merkle et al., 1998; Shourijeh et al., 2016b; Barroso et al., 2017). Synergies take a high dimensional control space and reduce it to a low dimensional space, which is potentially useful for reducing the level of indeterminacy when estimating muscle forces via optimization. Recent studies have demonstrated the potential utility of muscle synergies for facilitating motor learning in healthy and impaired individuals (d'Avella, 2016; Patel et al., 2017; Togo and Imamizu, 2017; Niu et al., 2019). Nonetheless, the use of muscle synergy information for neurorehabilitation remains controversial, as the muscle synergy hypothesis is difficult to prove or falsify (Tresch and Jarc, 2009; Kutch and Valero-Cuevas, 2012).

Several studies have used a synergy structure to reduce the dimensionality of the unknown muscle activation controls (Neptune et al., 2009; McGowan et al., 2010; Mehrabi et al., 2019). However, the models used in these studies were limited to sagittal plane motion and used a reduced number of muscles because the synergy information was extracted from electromyographic (EMG) measurements available from only superficial muscles. In contrast, a recent study applied a computational approach termed synergy optimization (SynO) to a three-dimensional walking model possessing 35 muscle–tendon actuators per leg, where each muscle could be associated with one of 16 experimentally measured surface or fine-wire EMG signals (Shourijeh and Fregly, 2020). The model's lower body joint motion and muscle–tendon force-generating properties were personalized to subject walking data using EMG-driven modeling approach (Meyer et al., 2017). The authors evaluated how the specified number of synergies affected estimated lower body joint stiffness and inverse-dynamics joint moment matching. While results obtained from SynO were compared with those obtained from SO, experimental evaluation of the muscle activations predicted by SynO was not performed. Furthermore, because imposition of a synergy structure on predicted muscle activations ties all time frames together, SynO is more complex and slower computationally than is SO.

This study evaluated whether imposition of a synergy structure on muscle activations estimated via inverse-dynamics optimization (i.e., SynO) produces muscle activation estimates that are more consistent with EMG measurements than are those produced by traditional SO. Muscle activations reconstructed by performing synergy analysis on SO activations were included in the evaluation as well. Muscle activations and inverse-dynamics joint moment matching from all three approaches were compared to activations derived from experimental EMG data and joint moments calculated by inverse dynamics using data collected from five subjects performing overground walking. Three-dimensional models of the subjects were used to perform the evaluation. Comparison of these three approaches provides insight into the extent to which, and the conditions under which, imposition of a synergy structure may improve the estimation of muscle forces during walking.

## Methods

### Experimental Data Collection

Five subjects (four males, one female; aged 42 ± 16 years; height 178 ± 11 cm; body mass 75 ± 25 kg) were recruited for this study. All subjects gave written informed consent for their participation. Subjects walked at their self-selected speed (1.1 ± 0.18 m/s) along a walkway with two embedded force plates (AccuGait, sampling at 100 Hz; AMTI, Watertown, MA, USA). The motion was captured using 12 optical infrared cameras (OptiTrack FLEX:V100, also sampling at 100 Hz; Natural Point, Corvallis, OR, USA) that computed the position of 37 optical markers. Additionally, 11 surface EMG signals on the right leg were recorded at 1 kHz (FREEEMG; BTS, Quincy, MA, USA). Each EMG signal was rectified, filtered by a singular-spectrum analysis with a window length of 250 (Romero et al., 2015) (equivalent to the common forward and reverse low-pass fifth-order Butterworth filter with a cutoff frequency of 15 Hz) and then normalized with respect to its maximal value as recommended in Raison et al. (2011). This cutoff frequency value is consistent with the ranges reported in previous studies using EMG data (Buchanan et al., 2004; Raison et al., 2011).

### Musculoskeletal Model Creation

**Figure 1:**
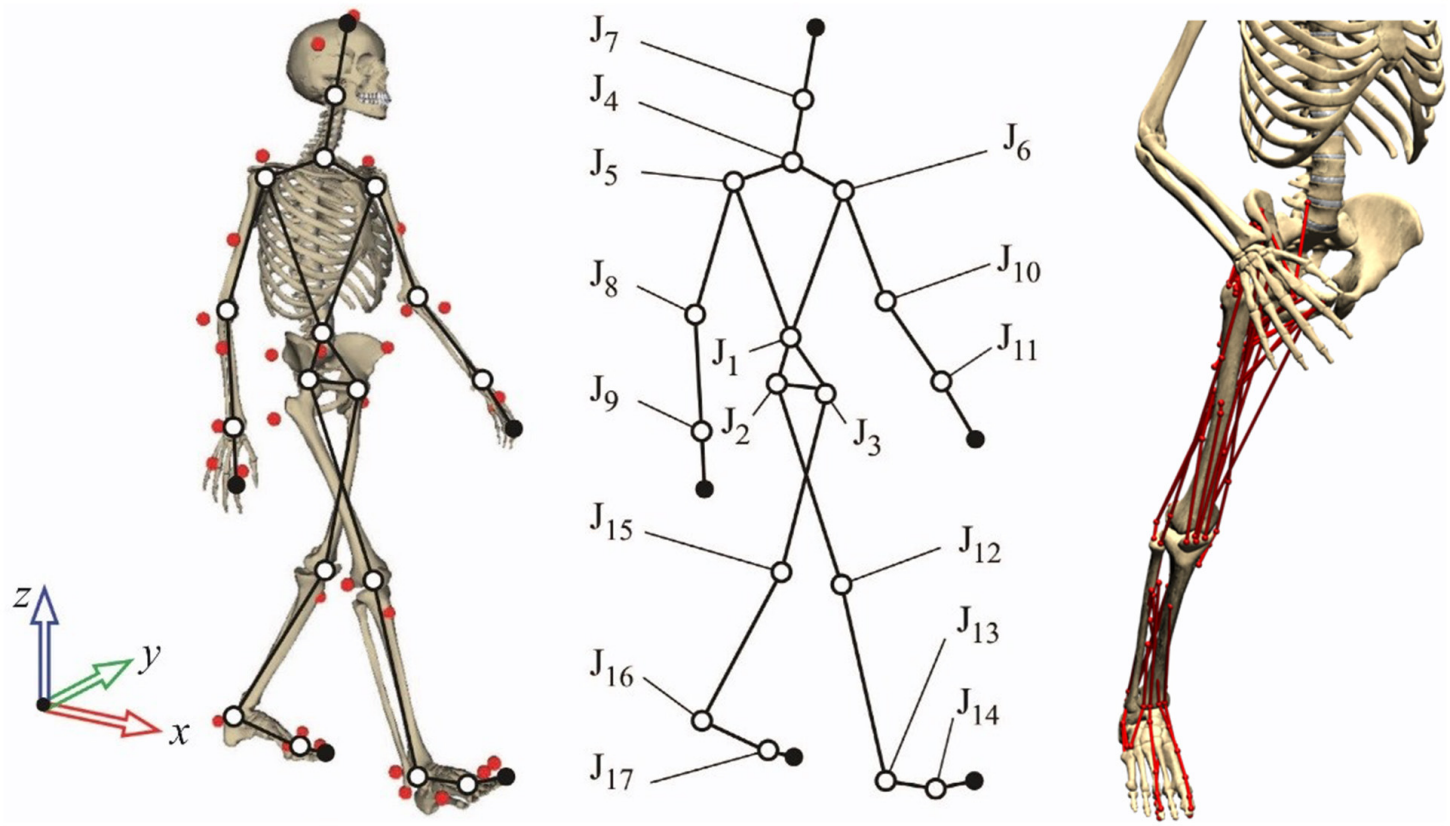
Three-dimensional human model and detail of muscles on the right leg.

The human body was modeled as a three-dimensional multibody system formed by rigid bodies (Figure 1, left and center). The model consisted of 18 anatomical segments (Lugrís et al., 2013b): two hindfeet, two forefeet, two shanks, two thighs, a pelvis, a torso, a neck, a head, two arms, two forearms, and two hands. The segments were linked by ideal spherical joints, thus defining a model with 57 DOFs. The axes of the global reference frame were defined as follows: *x*-axis in the anterior–posterior direction, *y*-axis in the medial–lateral direction, and *z*-axis in the vertical direction. The computational model was defined with 228 mixed (natural + angular) coordinates. The subset of natural coordinates comprised the three Cartesian coordinates of 22 points and the three Cartesian components of 36 unit vectors, thus yielding a total of 174 variables.

Matrix-R formulation (García de Jalón and Bayo, 1994) was applied to obtain the joint torques along the motion using the in-house–developed MBSLIM library (Dopico et al., 2016) programmed in FORTRAN, as described in Lugrís et al. (2013a). Once the joint torques were computed, we assumed that 43 right leg muscles contributed to six right leg inverse-dynamics moments: three rotational DOFs at the hip, the flexion/extension DOF at the knee, and the plantar/dorsi flexion and internal/external rotation at the ankle. Muscles were modeled as one or more straight-line segments with via points. These points corresponded to the attachments of muscle and tendon to bone and were defined as the origin (i.e., proximal attachment) and insertion (i.e., distal attachment). Muscle properties and local coordinates for these points were obtained from OpenSim (model Gait2392) (Delp et al., 2007) and scaled to each subject from the generic reference OpenSim model. Length parameters (optimal muscle fiber length and tendon slack length) were scaled, for each muscle, with a scale factor calculated as the relation between the subject's musculotendon length in a standing position and that of the generic model in the same position. Muscle forces were calculated from optimization-predicted muscle activations using a custom Hill-type rigid tendon–muscle model (Zajac, 1989) developed in MATLAB (MathWorks, Natick, MA, USA) (De Groote et al., 2016). For slow activities such as walking, use of a rigid tendon model is justifiable because it gives nearly identical muscle force estimates to those produced by a compliant tendon model (De Groote et al., 2016; Michaud, 2020). We assumed that not calibrating the positions and orientations of the joint functional axes in the leg model likely affected inverse-dynamics joint moment calculations (Reinbolt et al., 2007), which in turn likely affected muscle activation calculations. Moreover, not having a process for calibrating Hill-type muscle–tendon model properties likely affected the estimated muscle activations (Serrancolí et al., 2016). However, all the methods proposed in this work were used with the same limitations.

### Muscle Activation Estimation Approaches

Using this human body model, we explored three approaches for estimating muscle activations and quantified how closely each one reproduced experimental muscle EMG data. For the first approach, muscle activations were estimated using SynO. For the second approach, traditional SO was used. For the third approach, non-negative matrix factorization (NMF) was performed on the SO activations, and then muscle activation estimates were constructed from the synergies. For each approach, inverse-dynamics joint moment matching was quantified using the total variance account (VAF), whereas EMG matching was quantified via cross correlation using the Pearson correlation coefficient *r* (MATLAB's function *corrcoef*) with a maximum time delay of 100 ms (Shourijeh et al., 2016a). The correlation coefficient *r* was chosen to compare muscle activations and EMG data so as to focus on shape differences (between the activation patterns, the activation/no-activation areas) rather than magnitude differences, as there is no direct relationship between EMG amplitude and muscle force amplitude (Hof, 1997; Buchanan et al., 2004). Each of the three approaches for estimating muscle activations is described in greater detail below.

### Synergy Optimization

The SynO approach used in Shourijeh and Fregly (2020) estimates muscle forces during human walking using synergy-constructed muscle activations, similar to the more complex approach in Gopalakrishnan et al. (2014). Synergy optimization finds muscle forces that match the inverse-dynamics joint moments as closely as possible through the moment tracking error term in the cost function. In SynO, synergies couple muscle activations across time frames, requiring the optimization to be performed over all the time frames simultaneously as follows:

(1) $$\begin{array}{r} a_{f\text{xm}}=C_{f\text{x}n_{S}}\left(C_{p}\right)V_{n_{S}\times m} \end{array}$$

where *C*_*f*x_*n*__*S*__(*C*_*p*_) and *V*_*n*_*S*_×*m*_ are the time-varying synergy activations defined by B-spline nodes and the corresponding time-invariant synergy vectors, respectively. Each muscle activation synergy was composed of a single time-varying synergy activation defined by *p* = (*f* – 1)/5 + 1 (nearest integer, *f* = number of frames) B-spline nodal points along with its corresponding time-invariant synergy vector defined by *m* = 43 weights specifying intermuscle activation coupling. Thus, for *n*_*S*_ synergies (*n*_*S*_ = 2 through 6), the number of design variables was *n*_*S*_ × (*p* + *m*). Muscle synergy quantities were used as the design variables for SynO. On the other hand, the six joint moments multiplied by the *f* time frames led to 6*f* equations from inverse-dynamics joint moment matching. Therefore, the optimization problem was theoretically overdetermined. However, in practice, the problems remained underdetermined because neighboring time frames are not completely independent from one another.

Using these design variables, the SynO cost function was formulated as follows:

(2) $$\begin{array}{r} \underset{C_{p},V}{J_{SynO}}=\overset{n}{\underset{j=1}{\sum }}\left(\beta {\overset{6}{\underset{k=1}{\sum }}\left[\frac{Q_{jk}^{MT}-Q_{jk}^{ID}}{\max\left(\left|Q_{k}^{ID}\right|\right)}\right]}^{2}+\overset{m}{\underset{i=1}{\sum }}\left(a_{ij}^{2}+\lambda_{ij,pen}{\left(a_{ij}-1\right)}^{2}\right)\right) \end{array}$$

where *a*_*ij*_ is the synergy-based muscle activation, and $\lambda_{ij,pen}=\{\begin{array}{ll} 0 & 0\leq a_{ij}\leq 1\\ 10^{5} & \text{otherwise} \end{array}$ are penalization factors for muscle *i* at the time frame *j* to ensure that muscle activations stay between zero and one. β = 100 is a scale factor to give more importance to the minimization of the error between $Q_{k}^{ID}$, the vector of the inverse-dynamics joint moments for the *k*th DOF, and $Q_{k}^{MT}$, the joint moments produced by the muscle forces estimated by SynO. A broad range of β values (1, 10, 50, 100, 200, 500, and 1,000) was explored, and similar to Ou (2012), the best compromise between joint moment tracking and activation minimization was a value of 100.

The objective function was programmed as a Fortran mex file to reduce computation time (16 times faster than the original MATLAB function). Linear equality constraints made the sum of weights within each synergy vector equal to one, which made the synergy construction unique, whereas lower-bound constraints made the synergy activation B-spline nodes and synergy vector weights non-negative. Synergy optimization problems were solved using MATLAB's *fmincon* non-linear constrained optimization algorithm. Five global optimizations were run using MATLAB's *ga* genetic optimization algorithm with a population size of 50, providing random initial guesses for *fmincon*. The SynO's solution with the lowest objective function value was chosen as the final solution.

### Static Optimization

**Figure 2:**
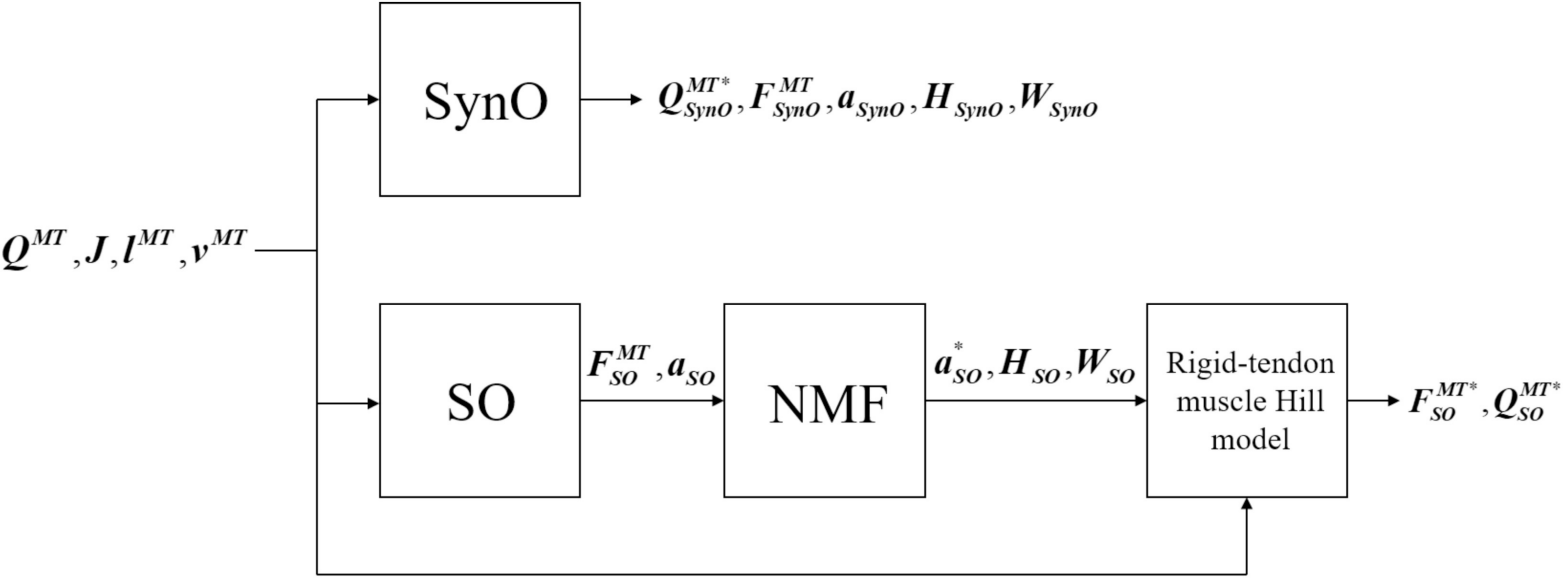
Block diagram of SynO and combined SO-NMF approaches. *Q*^*MT*^ is the vector of the intersegmental moments driven by muscles, *J* is the Jacobian matrix of moment arms, *l*^*MT*^and *v*^*MT*^ are, respectively, the length and velocity of the musculotendons. *F*^*MT*^, and *a* represent the estimated muscular forces and activations; *H*, the single time-varying synergy activation; and *W*, the time-invariant synergy vector. *Q*^MT*^, *F*^MT*^, and a^*^ are the reconstructed intersegmental moments, muscular forces, and activations.

In contrast to SynO, SO's muscle activations are independent between time frames, allowing the optimization to be performed one time frame at a time. Static optimization was run for the same conditions as SynO (Figure 2) using the same solver *fmincon* and carrying out five global optimizations to obtain the initial guess for the initial time point. Thereafter, as muscle activation is normally smooth and continuous during gait, the optimal solution from the previous time frame was used as the initial guess for the current time frame (e.g., Shourijeh et al., 2017). Unlike SynO, SO finds muscle forces that perfectly reproduce the inverse-dynamics joint moments (in the absence of reserve actuators) through equality constraints. Both optimization approaches were evaluated based on their ability to reproduce the inverse-dynamics joint moments and the shapes of the experimentally measured muscle excitations. In contrast to SynO, SO reproduces inverse-dynamics joint moments perfectly through its equality constraints, which can be viewed as a high-penalty weight in an unconstrained optimization cost function.

### Identification of Muscle Synergies From Static Optimization

To extract a synergy structure from the SO results, we used NMF to decompose the 43 muscles activations estimated by SO:

(3) $$\begin{array}{r} a^{*}=\overset{n}{\underset{i=1}{\sum }}\left(W_{i}\times H_{i}\right) \end{array}$$

where a^*^ is the vector of the reconstructed muscular activations, *H*_*i*_ is the single time-varying synergy activation, and *W*_*i*_ is the corresponding time-invariant synergy vector for each of the *n* synergies (*n* = 2 through 6). MATLAB *nnmf* was modified to constrain the norm-1 of each synergy vector to one to have the same constraint as SynO. Finally, using the rigid tendon Hill-type muscle model, the reconstructed muscle forces and corresponding intersegmental joint moments were derived from a^*^. This approach was called SO-NMF in this work.

In what follows, it will be first checked that muscles produce acceptable joint moments, and then the three different approaches will be evaluated by comparing the predicted muscle activations obtained with experimental EMG data.

## Results

**Figure 3:**
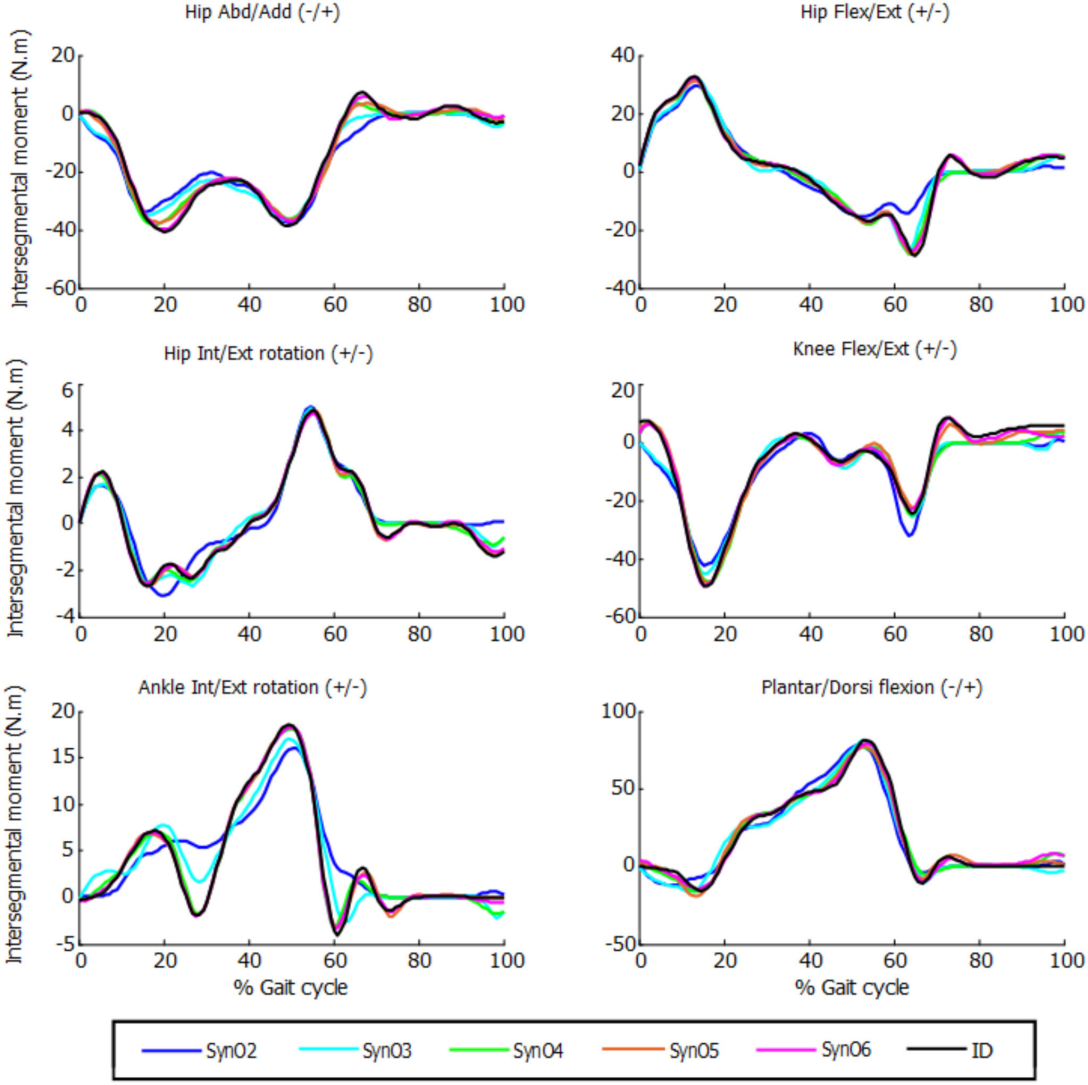
Intersegmental moments from SynO for *n* synergies (*n* = 2 through 6) vs. intersegmental moments calculated by inverse dynamics for one subject.

The joint moments obtained from SynO using two through six synergies matched the inverse-dynamics joint moments well (Table 1, Figure 3). The worst match was produced when using only two synergies, although the model was still able to match the inverse-dynamics joint moments closely (mean VAF of 85%). With three synergies, the mean VAF obtained was higher than 96% for all the subjects. Between four and six synergies, VAF values were 98% or higher.

**Table 1:** Mean correlation VAF values across subjects between intersegmental moments calculated by inverse-dynamics and (i) joint intersegmental moments from SynO, (ii) joint intersegmental moments from NMF with SO, for *n* synergies (*n* = 2 through 6) for the five subjects.

	**Mean VAF values across subjects for joint intersegmental moment matching**										
	**Two synergies**		**Three synergies**		**Four synergies**		**Five synergies**		**Six synergies**		**SO**
	**SynO**	**SO-NMF**	**SynO**	**SO-NMF**	**SynO**	**SO-NMF**	**SynO**	**SO-NMF**	**SynO**	**SO-NMF**	
Hip abd/add.	91.25	73.36	96.63	86.19	98.91	85.31	99.39	92.59	99.79	92.73	100.00
Hip flex/ext.	92.99	78.16	97.90	94.29	98.95	95.13	99.70	93.76	99.80	97.67	100.00
Hip int/ext rot.	94.45	24.99	97.83	26.84	98.80	35.75	99.73	54.08	99.85	49.05	100.00
Knee flex/ext.	92.80	35.61	96.42	52.74	98.19	88.62	99.38	87.62	99.70	92.50	100.00
Ankle int/ext rot.	75.59	52.64	91.99	51.59	98.29	68.74	99.81	80.12	99.87	85.59	100.00
Plantar/dorsi flex.	91.87	75.04	95.80	80.70	98.17	84.98	98.93	91.69	99.69	91.02	100.00
Mean across joints	89.82	56.63	96.10	65.39	98.55	76.42	99.49	83.31	99.78	84.76	100.00

*(VAF <95% is considered not good enough, in red)*.

**Figure 4:**
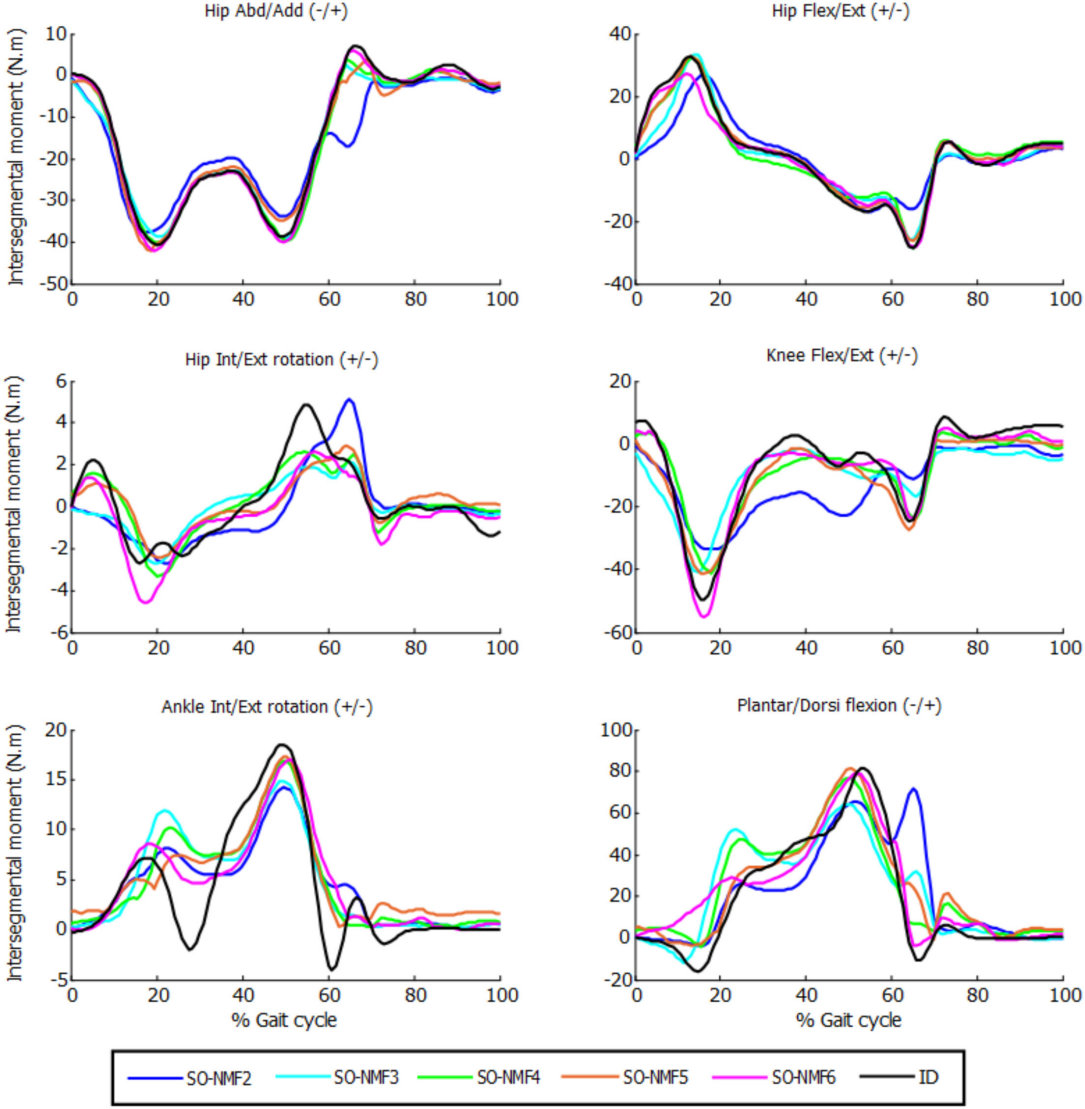
Intersegmental moments from SO and NMF for *n* synergies (*n* = 2 through 6) vs. intersegmental moments calculated by inverse dynamics for one subject.

While SO exactly reproduced the inverse-dynamics joint moments through its equality constraints, SO-NMF's muscular activations with two through six synergies matched the experimental inverse-dynamics joint moments poorly (Table 1, Figure 4). With two and three synergies, matches for some joint moments were worse than 50% VAF, and the mean match was lower than 70%. Between four and six synergies, mean VAF values were between 76% (with four synergies) and 90% (with six synergies), and some joint moments remained <80%.

**Figure 5:**
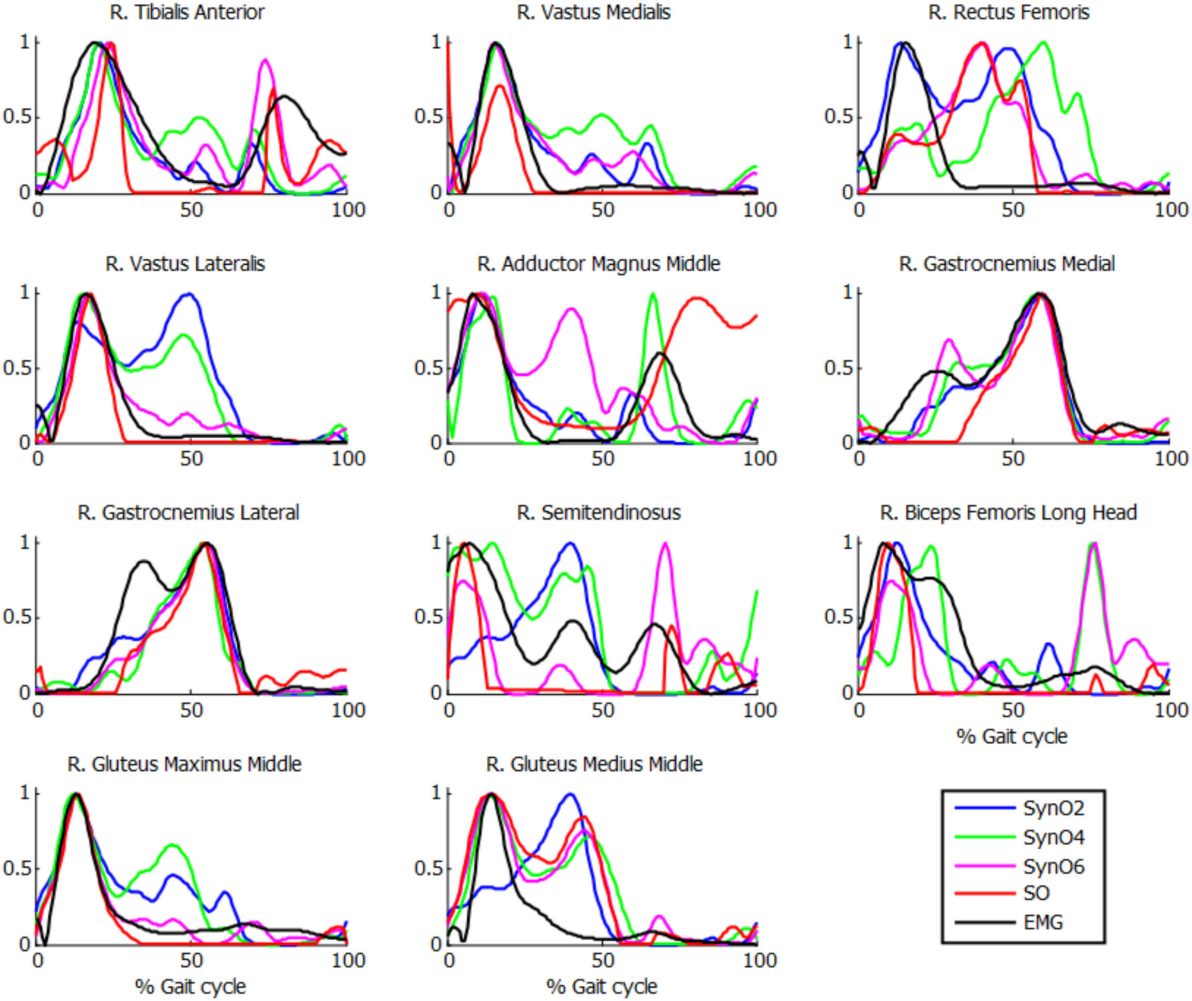
Normalized muscle activations obtained for one subject from SynO and *n* synergies (*n* = 2 through 6) vs. normalized EMG.

Comparison of muscle activations estimated using SynO with experimental EMG measurements showed significant differences when the number of synergies was increased (example in Figure 5 for one of the subjects). Activations estimated by SynO became more similar to those estimated by SO as the number of synergies was increased. However, the mean correlations *r* between estimated muscle activations and measured EMG patterns for the five subjects did not present such differences (Table 2). Mean values of the different approaches were close, between 0.56 (four synergies) and 0.62 (six synergies) for SynO and 0.60 for SO.

**Table 2:** Mean across subjects correlation coefficient *r* values between EMG measurements and (i) muscular activations from SynO, (ii) muscular activations from NMF with SO, for *n* synergies (*n* = 2 through 6) of the five subjects (*r* < 0.4, in red, is considered poor, and *r* ≥ 0.6, in green, is considered good).

	**Pearson correlation coefficient** ***r*** **between across-subject mean EMG vs. muscle activations**										
	**Two synergies**		**Three synergies**		**Four synergies**		**Five synergies**		**Six synergies**		**SO**
	**SynO**	**SO-NMF**	**SynO**	**SO-NMF**	**SynO**	**SO-NMF**	**SynO**	**SO-NMF**	**SynO**	**SO-NMF**	
R. tibialis anterior	0.58	0.52	0.59	0.75	0.56	0.75	0.56	0.74	0.69	0.76	0.69
R. vastus medialis	0.84	0.74	0.61	0.84	0.68	0.74	0.68	0.75	0.73	0.76	0.71
R. rectus femoris	0.53	−0.07	0.52	−0.09	0.25	−0.02	0.08	−0.06	0.16	−0.05	−0.04
R. vastus lateralis	0.72	0.74	0.75	0.82	0.65	0.80	0.54	0.78	0.73	0.79	0.74
R. adductor magnus middle	0.48	0.54	0.43	0.71	0.52	0.51	0.57	0.56	0.57	0.56	0.52
R. gastrocnemius medial	0.72	0.80	0.87	0.71	0.77	0.67	0.70	0.69	0.75	0.67	0.60
R. gastrocnemius lateral	0.57	0.72	0.76	0.77	0.67	0.66	0.70	0.70	0.64	0.71	0.57
R. semitendinosus	0.36	0.73	0.58	0.89	0.53	0.66	0.66	0.67	0.50	0.60	0.57
R. biceps femoris long head	0.72	0.69	0.57	0.85	0.51	0.79	0.50	0.80	0.54	0.86	0.84
R. gluteus maximus middle	0.74	0.71	0.71	0.86	0.71	0.90	0.84	0.92	0.87	0.92	0.91
R. gluteus medius middle	0.25	0.39	0.45	0.41	0.36	0.40	0.48	0.42	0.38	0.43	0.44
Mean	0.59	0.60	0.62	0.68	0.56	0.62	0.57	0.64	0.59	0.64	0.60

**Figure 6:**
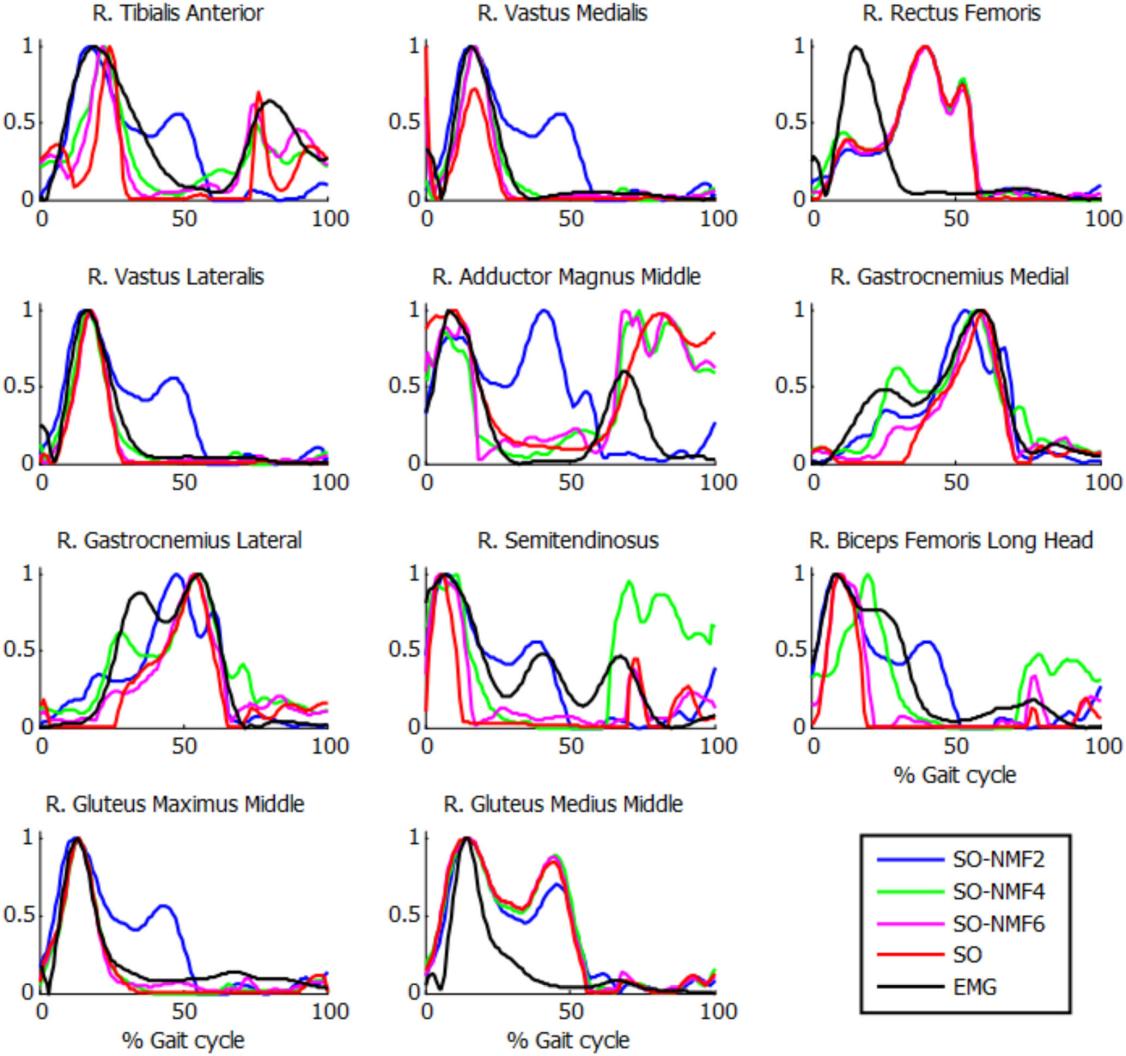
Normalized muscle activations obtained for one subject from SO and NMF with *n* synergies (*n* = 2 through 6) vs. normalized EMG.

Reconstructed muscle activations obtained using SO-NMF poorly matched the activations estimated using SO (Table 3, Figure 6). Using only two synergies, a mean *r*^2^ correlation of 0.44 was obtained for the 43 muscles, and a maximum correlation of 0.87 was obtained with six synergies. However, while reconstructed muscle activations and reconstructed joint moments showed low correlations with SO results, correlations between experimental EMG patterns and the newly reconstructed activations showed better mean values. The best correlations were obtained using three synergies, with a mean value of 68%. From two to six synergies, the correlations varied between 60 and 68%, giving similar or better results than those obtained using SO estimated activations.

**Table 3:** Mean correlation coefficient *R*^2^-values between muscular activations calculated by SO and SO-NMF for *n* synergies (*n* = 2 through 6).

	***r***^****2****^ **Mean values between SO and SO-NMF**				
	**Two synergies**	**Three synergies**	**Four synergies**	**Five synergies**	**Six synergies**
*a* vs. a^*^	0.44	0.56	0.75	0.83	0.87

The extracted synergies were significantly different between SynO and SO-NMF. With a reduced number of synergies, the SO-NMF method poorly reproduced the muscle activations calculated by SO (Table 3) by prioritizing muscles with higher activations, whereas SynO offered a better correlation and more homogenous solution.

Finally, the computational efficiency of the different approaches studied in this work was compared in Table 4. All calculations were performed on an Intel® Core™ i7-6700K processing running at 4.00 GHz with 16 GB of RAM, and all functions (except the objective function of SynO programmed in a *mex* file) were programmed in MATLAB using the optimization function *fmincon* without parallelization. Computation time increased significantly with the number of synergies and with SO clearly being the fastest method, requiring a mean duration of 2 s to solve a complete gait cycle of ~1 s. The NMF analysis required ~1 s in MATLAB.

**Table 4:** Mean computational time for SO and SynO with *n* synergies (*n* = 2 through 6) of the five subjects.

	**SynO2**	**SynO3**	**SynO4**	**SynO5**	**SynO6**	**SO**
Computational time (s)	80	115	196	317	587	2

## Discussion

This work analyzed whether a recent synergy-based approach used to solve the muscle force sharing problem, called SynO (Shourijeh and Fregly, 2020), can improve estimation of muscle activations during gait. In addition to comparing the correlations between estimated activations obtained by SO and SynO for five healthy subjects, we explored the reliability of predicting muscle activations by applying NMF to SO's muscle activations. Increasing the number of synergies from two until six in SynO had minimal influence on the model's ability to match inverse-dynamics joint moments closely. On the other hand, reconstructed joint moments from SO combined with NMF matched inverse-dynamics joint moments poorly, because unlike SynO, NMF does not take into account any joint moment information. Consequently, the resulting joint moments would produce a new motion, different from the original one.

Muscle activations obtained from SynO using two through six synergies exhibited visually different shapes, as reported previously by Shourijeh and Fregly (2020). Increasing the number of synergies implies increasing the number of design variables, thus allowing more freedom in the behavior of muscle activations. For this reason, SO presented results closer to SynO with six synergies. The same observations can be made with NMF when varying the number of synergies (Figure 6).

The highest muscle activations were observed for two synergies (blue line), which generated higher co-contraction when seeking to match the intersegmental moments, which would likely produce higher joint stiffness (Shourijeh and Fregly, 2020). Individuals with neurological disorders such as stroke or Parkinson's disease often use a lower number of muscle synergies than do healthy individuals (Clark et al., 2009; Rodriguez et al., 2013). Consequently, individuals with these disorders may generate higher stiffness to maintain stability and reject walking disturbances (Rinalduzzi et al., 2015; Kitatani et al., 2016).

Correlations observed in Table 2 are reasonable in general, with mean *r* values for the five subjects varying between 0.56 and 0.68. Surprisingly, no significant differences were observed for different numbers of synergies. The poorest results were obtained for the rectus femoris and the gluteus medius. Crosstalk (Jungtäubl et al., 2018) may explain the low correlation for these muscles, especially rectus femoris. Comparing the rectus femoris EMG signal with the vastus intermedius (muscle located under the rectus femoris) estimated activation resulted in a higher correlation (from 0.25 to 0.61). Furthermore, EMG correlations produced by SynO (two through six synergies) were essentially the same as than those produced by SO. Despite its higher dimensional control space, SO produced a mean correlation coefficient of 0.60, whereas SynO correlations ranged from 0.56 to 0.62.

Strangely, the reconstructed activations from SO-NMF matched EMG better than did the original activations from SO. However, the reconstructed inverse-dynamics joint moments showed a poor correlation VAF (between 56 and 85%), thus producing an inconsistent actuation. This might have been caused by the use of a reduced number of components when obtaining the synergy information through NMF for a large number of muscles.

Synergy optimization constructs the activation from optimized synergy activations (C) and synergy weights vectors (V) (Equation 1), whereas NMF, by definition, decomposes a signal into C and V. Consequently, the extracted synergies and reconstructed activations were significantly different between SynO and SO-NMF.

For SynO as well as for SO-NMF, the best correlations with experimental EMG patterns were obtained using three synergies. As mean intersegmental moment matching with three synergies was good using SynO (96.1% in Table 1, although the matching of the internal/external rotation moment at the ankle was only 92.0%), it appears that the CNS could control one leg during gait using only three synergies. Olree and Vaughan (1995) recorded EMG signals bilaterally from eight leg muscles and also showed that three basic patterns could account for the locomotion activity of these muscles. However, based on EMG activity analysis of 16 unilateral leg muscles (Winter and Yack, 1987), Davis and Vaughan (1993) and Ivanenko et al. (2004) concluded, respectively, that four and five patterns could be necessary. As explained in Banks et al. (2017) and Steele et al. (2013), variations in methodological choices, as unilateral or bilateral analysis, selected muscles, EMG processing, or computational method, may generate different results. Therefore, it is difficult to conclude what number of synergies is used by the CNS during gait. In this work, although only one leg was studied, it would be interesting to explore how many bilateral synergies would be found using SynO when studying both legs together, especially in the case of unilateral stroke (Sainburg et al., 2013; Coscia et al., 2015).

In conclusion, this study evaluated the ability of the SynO approach to predict muscle activations obtained from experimental EMG measurements during gait and found that three synergies are theoretically enough to control leg muscles during gait. However, no significant differences in ability to predict experimental EMG patterns were found between SynO with *n* synergies (*n* = 2 through 6) and SO; thus, neither approach can be considered preferable for this purpose. While SO is computationally faster and requires muscle forces to match inverse-dynamics joint moments through constraints, extraction of synergies by NMF from SO's results generated new intersegmental joint moments that were inconsistent with the experimental joint moments. Because the use of synergy structure does not show improvements with respect to the commonly used SO, observations made by Kutch and Valero-Cuevas (2012) could explain our results. The SynO approach offers reasonable prediction of muscle activations using an imposed synergy structure and reduced dimensional control space and could be useful for applications such as functional electrical stimulation and motion control and prediction.

## Data Availability Statement

The datasets generated for this study are available on request to the corresponding author.

## Ethics Statement

The studies involving human participants were reviewed and approved by the Committee of Ethics of the University of A Coruña. The patients/participants provided their written informed consent to participate in this study.

## Author Contributions

BF and MS developed the SynO approach. FM designed and performed the experiments, derived the models, and analyzed the data with the supervision of BF, MS, and JC. FM, BF, MS, and JC wrote the manuscript. All authors contributed to the article and approved the submitted version.

## Conflict of Interest

The authors declare that the research was conducted in the absence of any commercial or financial relationships that could be construed as a potential conflict of interest.
